# Relationship between the Use of Inhaled Steroids for Chronic Respiratory Diseases and Early Outcomes in Community-Acquired Pneumonia

**DOI:** 10.1371/journal.pone.0073271

**Published:** 2013-09-05

**Authors:** Jordi Almirall, Ignasi Bolíbar, Mateu Serra-Prat, Elisabet Palomera, Jordi Roig, Imma Hospital, Eugenia Carandell, Mercè Agustí, Pilar Ayuso, Andreu Estela, Antoni Torres, and the Community-Acquired Pneumonia in Catalan Countries (PACAP)

**Affiliations:** 1 Critical Care Unit, Universitat Autònoma de Barcelona, CIBERES, Barcelona, Spain; 2 Department of Clinical Epidemiology and Public Health, Institute of Biomedical Research (IIB Sant Pau) Barcelona, Universitat Autònoma de Barcelona, Ciber de Epidemiología y Salud Pública (CIBERESP); 3 Research Unit, Hospital de Mataró, CIBEREHD, Barcelona; 4 Hospital Nostra Senyora de Meritxell, Principat d’Andorra; 5 Institut Català de la Salut (ICS), Basrcelona; 6 IB-SALUT Balears, Palma de Mallorca; 7 INSALUD, Valencia, Spain; 8 Servei de Pneumologia, Institut Clínic del Torax, IDIBAPS, Hospital Clínic de Barcelona, Universitat de Barcelona, CIBERES, Barcelona, Spain; D'or Institute of Research and Education, Brazil

## Abstract

**Background:**

The role of inhaled steroids in patients with chronic respiratory diseases is a matter of debate due to the potential effect on the development and prognosis of community-acquired pneumonia (CAP). We assessed whether treatment with inhaled steroids in patients with chronic bronchitis, COPD or asthma and CAP may affect early outcome of the acute pneumonic episode.

**Methods:**

Over 1-year period, all population-based cases of CAP in patients with chronic bronchitis, COPD or asthma were registered. Use of inhaled steroids were registered and patients were followed up to 30 days after diagnosis to assess severity of CAP and clinical course (hospital admission, ICU admission and mortality).

**Results:**

Of 473 patients who fulfilled the selection criteria, inhaled steroids were regularly used by 109 (23%). In the overall sample, inhaled steroids were associated with a higher risk of hospitalization (OR=1.96, *p* = 0.002) in the bivariate analysis, but this effect disappeared after adjusting by other severity-related factors (adjusted OR=1.08, p=0.787). This effect on hospitalization also disappeared when considering only patients with asthma (OR=1.38, p=0.542), with COPD alone (OR=4.68, p=0.194), but a protective effect was observed in CB patients (OR=0.15, p=0.027). Inhaled steroids showed no association with ICU admission, days to clinical recovery and mortality in the overall sample and in any disease subgroup.

**Conclusions:**

Treatment with inhaled steroids is not a prognostic factor in COPD and asthmatic patients with CAP, but could prevent hospitalization for CAP in patients with clinical criteria of chronic bronchitis.

## Introduction

Community-acquired pneumonia (CAP) remains an important cause of morbidity and mortality in industrialised countries, with a hospitalization rate between 22% and 51% [1,2] and a lethality rate between 3% and 24% [3,4]. Chronic bronchitis (CB), chronic obstructive pulmonary disease (COPD) and asthma are well known risk factors for CAP [4–6]. Some studies have suggested that treatment with inhaled steroids may also increase the risk of CAP, the effect of which being independent of the underlying diseases suffered by these patients [7,8]. In The TOwards a Revolution in COPD Health (TORCH) study, there was an increased probability of having pneumonia reported as an adverse event in patients treated with inhaled fluticasone propionate either alone or in combination with salmeterol compared with patients randomised to salmeterol alone or placebo (7). Further studies have also shown an association of inhaled steroids therapy and the incidence of CAP in COPD patients [8–11]. However, despite the increase in the frequency of pneumonia observed with the use of inhaled steroids in chronic pulmonary disease, there has been no associated rise in mortality reported by any of the recent trials [7–9,12]. On the contrary, some authors have shown a reduction of mortality in COPD patients treated with inhaled steroids [12,13]. This had led to speculation that the use of inhaled steroids may increase the risk for CAP but protect against severe pneumonia or pneumonia-related complications [14,15].

The present study aimed to explore whether treatment with inhaled steroids in patients with CB, COPD or asthma who developed CAP may affect the prognosis of the acute pneumonic episode.

## Methods

### Design and study population

The Community-Acquired Pneumonia in Catalan Countries (PACAP) study is a population-based study conducted in an extensive rural and urban area on the Eastern Coast of Spain, with predominantly Mediterranean climatic conditions, aimed to identify risk factors for CAP. Over 1-year period, 1336 incident cases of CAP from a population of 859,033 inhabitants > 14 years of age were recruited. Details of this study have been previously published elsewhere [16].

### Identification of cases

All suspected cases of CAP occurring in the study population between November 1999 and November 2000 were registered. Predefined criteria for case registration were based on acute lower respiratory tract infection for which antimicrobials had been prescribed in association with the appearance of previously unrecorded focal signs on physical examination of the chest and new radiologic findings suggestive of pulmonary infiltrate [17]. All cases in which criteria for clinical suspicion were met were periodically re-evaluated until complete recovery. Patients in whom the diagnosis was not confirmed because of clinical evolution and chest radiograph images not consistent with CAP were excluded as were patients with aspiration pneumonia, active pulmonary tuberculosis, and patients coming from nursing homes or having been discharged from hospital < 7 days before the onset of symptoms. All public and private medical centres of the study area as well as reference hospitals outside the area participated in the reporting of cases.

The present study was restricted to patients with CAP who were candidates to use inhaled steroids due to the following baseline chronic respiratory disease: chronic bronchitis, COPD or asthma. From the original study population of 1,336 cases of CAP, 473 presented with chronic bronchitis, COPD or asthma.

The study protocol was approved by the Institutional review board of the *Consorci Sanitari del Maresme* (Barcelona, Spain) and all participants gave written informed consent before enrollment.

### Data collection and main outcome measures

At the time of CAP diagnosis, a questionnaire on CAP risk factors was administered directly to participants by trained physicians or nurses at home. The questionnaire included standardised information related to the following three aspects relating prognosis and severity of CAP: 1) health habits and lifestyle, 2) chronic respiratory diseases and other clinical conditions and comorbidity, including the CRB-65 severity score [18] for the current pneumonic episode, and 3) regular treatments during the last year. COPD was defined by the presence of persistent airflow limitation diagnosed by respiratory function tests documented in the medical records or stated by the patient. Patients with chronic bronchitis did not have spirometric study or COPD and were defined by its arbitrary epidemiological characterization of cough and expectoration over 90 days per year in two consecutive years and not secondary to any specific respiratory disease [19]. Asthma was defined by the presence of episodes of validated clinical symptoms and/or confirmatory medical documentation of its diagnosis [20]. Treatments were confirmed by medical records, prescriptions or, when necessary, by direct observation.

All patients were followed during 30 days after CAP diagnosis and/or until complete healing or death. Main outcome variables for CAP included hospital admission, length of hospital stay, ICU admission, 30-day mortality percentage and number of days to clinical healing (disappearance of all clinical symptoms).

### Statistical analysis

The bivariate effect of inhaled steroids on hospital admission, ICU admission and mortality was estimated as odds ratio (OR) and the 95% confidence interval (CI) by logistic regression analysis. In addition to the crude bivariate analysis for hospital admission, a multivariable analysis was performed in which the effect of inhaled steroids was adjusted by Propensity Score. The Propensity Score, was calculated by a multivariate logistic regression for inhaled steroids, with all variables associated with inhaled steroids in the bivariate analysis, (sex, age, hospital admission in the last 5 years, home oxygen therapy, previous radiologically-confirmed pneumonia, oral steroids and alcohol consumption). This model gives an individual’s probability of being treated with inhaled steroids given the complete information about that individual. This probability was used to adjust the effect of inhaled steroids on hospital admission. The effect of inhaled steroids on days of hospital stay and days until clinical recovery/healing was estimated by the beta coefficient (β) and its 95% CI of the linear regression model. Finally, survival analysis was performed with the Kaplan-Meier method. Survival curves for the event of “clinical healing” for the groups with and without inhaled steroids were compared with the log-rank test. All these analyses were performed for the overall sample and for the following subgroups: patients with asthma alone, patients with CB alone and patients with COPD alone. Statistical significance was set at *p*< 0.05.

## Results

A total of 473 patients with CAP confirmed by clinical evolution and radiographic findings with history of CB, COPD or asthma were identified. Sixty percent of patients were men with a mean age of 62 years and 40% women with a mean age of 55 years. Use of inhaled steroids was recorded in 109 (23%) patients. Hospital admission was required in 226 (47.8%) patients and ICU admission in 19 (4.0%) patients. A total of 10 patients died, with a 30-day mortality percentage of 2.1%.

**Figure 1:**
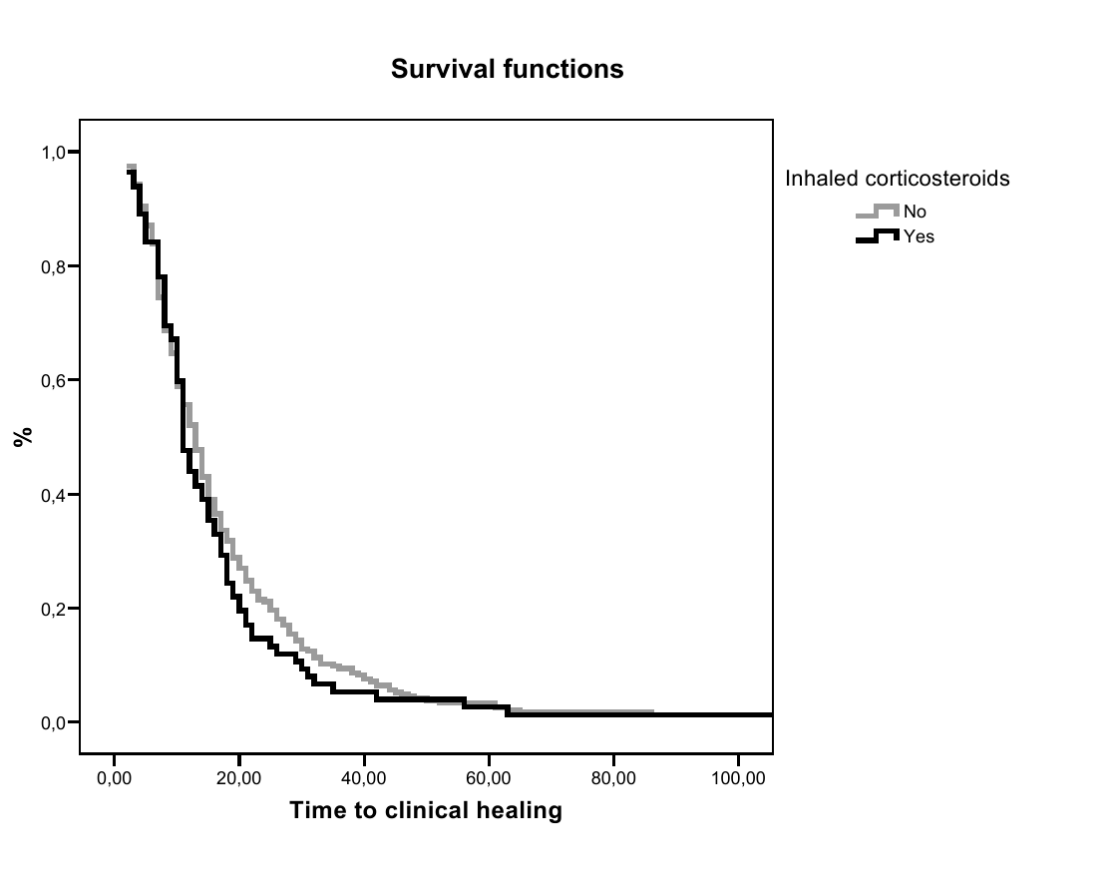
Time to clinical healing according to the use of inhaled steroids in patients with CAP.

The association between treatment with inhaled steroids and the different CAP outcomes for the overall sample and for the three study groups are shown in Table 1. In the overall sample, treatment with inhaled steroids was significantly associated with hospital admission, doubling the estimated risk of admission in relation to the patients without treatment. However, there were no significant relationships between inhaled steroids and ICU admission, 30-day mortality, length of hospital stay or days to clinical healing. In the Kaplan-Meier analysis, time to clinical healing of the pneumonic episode for patients with and without treatment with inhaled steroids was also similar (log-rank 0.416) (Figure 1). In those patients with known doses of inhaled steroids (n=55) we observed a dose–response trend with the risk of hospital admission (OR=4.62 for using 1-2 puffs/day, OR=8.93; p<0.001). The stratified analysis by type of respiratory disease showed that patients with clinical signs or symptoms of CB, inhaled steroids nearly reach a significant protective effect on CAP hospital admission, and patients with COPD, had a significant risk for CAP hospital admission.

**Table 1:** Association between treatment with inhaled steroids and main outcomes in patients with CAP.

Outcome variables	Use of inhaled steroids		Effect estimation	*p* value
	Yes No. (%)	No No. (%)		
**TOTAL**	(n = 109)	(n = 364)		
Hospital admission	66 (60.0)	160 (44.0)	1.96^*^ (1.27-3.03)	0.002
ICU admission	6 (5.5)	13 (3.6)	1.57^*^ (0.58-4.24)	0.404
Death (30-day mortality)	2 (1.8)	8 (2.2)	0.81^*^ (0.17-3.90)	1.000
Length hospital stay, days, mean ± SD	9.0 ± 4.9	8.9 ± 6.0	0.09^†^ (-1.55-1.73)	0.380
Days to clinical recovery, mean ± SD	14.4 ± 10.9	16.1 ± 12.7	-1.71^†^ (-4.80-1.38)	0.497
**ASTHMA**	(n=22)	(n=234)		
Hospital admission	10 (45.5)	78 (33.3)	1.67 (0.69-4.03)	0.252
ICU admission	1 (4.5)	7 (3.0)	1.54 (0.18-13.2)	0.518
Death (30-day mortality)	1 (5.3)	3 (1.5)	3.69 (0.36-37.3)	0.304
Length hospital stay, days, mean ± SD	10.7 (7.1)	8.5 (5.6)	2.16 (-1.71-6.03)	0.368
Days to clinical recovery, mean ± SD	15.1 (11.3)	15.9 (11.7)	-0.76 (-7.14-5.62)	0.751
**CB**	(n=12)	(n=26)		
Hospital admission	5 (41.7)	20 (76.9)	0.21 (0.05-0.93)	0.064
ICU admission	0 (0)	0 (0)	—	—
Death (30-day mortality)	0 (2)	2 (8.7)	—	1.000
Length hospital stay, days, mean ± SD	8.6 (3.5)	8.7 (5.3)	-0.14 (-5.37-5.10)	0.774
Days to clinical recovery, mean ± SD	14.2 (7.7)	14.8 (11.3)	-0.54 (-9.00-7.93)	0.667
**COPD**	(n=22)	(n=25)		
Hospital admission	21 (95.5)	17 (68.0)	9.88 (1.12-87.0)	0.025
ICU admission	1 (4.5)	0 (0)	—	0.468
Death (30-day mortality)	0 (0)	0 (0)	—	—
Length hospital stay, days, mean ± SD	7.5 (3.7)	8.2 (6.5)	-0.66 (-4.11-2.78)	0.939
Days to clinical recovery, mean ± SD	9.4 (7.8)	15.6 (20.2)	-6.21 (-16.3-3.88)	0.456

* Odds ratio and 95% confidence interval from logistic regression.

† Regression coefficients and 95% confidence intervals from linear regression.

The treatment with inhaled steroids had a relationship with most factors related to prognosis and severity of CAP (Table 2). Hence, the use of inhaled steroids was significantly associated with age, home oxygen therapy, hospital admission in the past 5 years, previous pneumonia confirmed by radiological findings, oral steroids, alcohol consumption, and CRB-65 score. Most of these factors (age, home oxygen therapy, hospital admission in the past 5 years, oral steroids) appeared associated with the need of hospital admission of patients with CAP, but not for ICU admission nor for 30-day mortality.

**Table 2:** Association between use of inhaled steroids and factors related to prognosis and severity of CAP.

	Use of inhaled steroids		OR (95% CI)	*p* value
	Yes (n = 109)	No (n = 364)		
Sex (% females)	41 (37.6)	148 (40.7)	0.88 (0.57-1.37)	0.569
Age, years, mean ± SD	68.3 ± 17.1	56.9 ± 20.1	1.03 (1.02-1.05)	< 0.001
Number of affected lobes > 1	9 (8.9)	33 (9.6)	0.92 (0.43-2.0)	0.837
Home oxygen therapy	15 (14.6)	13 (4.0)	4.14 (1.90-9.03)	< 0.001
Hospital admission in the last 5 years	79 (72.5)	177 (48.6)	2.78 (1.74-4.44)	< 0.001
Number hospital admissions in the last 5 years, mean ± SD	3.6 ± 3.6	2.2 ± 2.8	1.17 (1.05-1.31)	< 0.001
Previous radiologically-confirmed pneumonia	38 (34.9)	75 (20.6)	2.06 (1.29-2.30)	0.002
Number of previous radiologically-confirmed CAP, mean ± SD	0.6 ± 1.0	0.3 ± 0.7	1.59 (1.26-2.07)	0.001
Oral steroids	25 (22.9)	10 (2.7)	10.5 (4.87-22.8)	< 0.001
Smoking habit (never smoker)	43 (39.4)	114 (31.3)	1.43 (0.92-2.23)	0.114
Alcohol consumption	3 (2.8)	36 (9.9)	0.26 (0.08-0.85)	0.017
CBR-65 score (moderate/high risk of death)	28 (44.4)	44 (23.7)	2.58 (1.42-4.71)	0.002

Odds ratio and 95% confidence interval.

Data as absolute numbers and percentages in parenthesis unless otherwise stated.

Table 3 shows, for the overall sample and for the three study groups, the effect of inhaled steroids on hospitalization for CAP adjusted by Propensity Score. Treatment with inhaled steroids was not associated with hospitalization for CAP in the overall sample and in patients with asthma, COPD and COPD with hiperreactivitat bronquial, but showed a protective effect in patients with CB. In patients with COPD a strong effect increasing the risk of hospitalization was observed although it did not reach the statistical significance. The adjusted effect of inhaled steroids on ICU admission and mortality continued being not statistically significant. For continuous outcomes, i.e. number of days of hospital stay and number of dais to clinical healing, multiple linear regression models neither show a statistically significant independent effect of inhaled steroids.

**Table 3:** Effect of treatment with inhaled steroids on hospitalization for CAP adjusted by potential confounding factors.

**Hospital admission**	Effect estimation^*^	*p* value
**TOTAL**		
Inhaled steroids	1.08 (0.64-1.81)	0.787
**ASTHMA**		
Inhaled steroids	1.38 (0.49-3.87)	0.542
**CB**		
Inhaled steroids	0.15 (0.03-0.80)	0.027
**COPD**		
Inhaled steroids	4.68 (0.46-47.8)	0.194

* Odds ratio and 95% confidence interval from logistic regression adjusted by Propensity Score.

## Discussion

It has been suggested that treatment with inhaled steroids may prevent a higher severity of CAP in relation to reduction of airway inflammation and recruitment of neutrophils, leading to a blunted systemic inflammatory response [21,22]. Clinically, this has been analysed in different studies, such as the TORCH study [7] in which a lower mortality in COPD patients with CAP previously treated with inhaled steroids was found. This observation was also reported in the INSPIRE study [12]. In this study, although there was a greater risk of CAP in the combined treatment arm (salmeterol and fluticasone) as compared with tiotropium bromide, the mortality after 2 years was lower (3% vs. 6%, *P* < 0.032). In this respect, Malo de Molina et al. [13] also demonstrated a reduction of 24% in the 30-day mortality rate associated with the use of inhaled steroid therapy in COPD patients older than 64 years of age admitted to the hospital because of CAP. A lower mortality was not found in the study of Kardos et al. [9] when salmeterol monotherapy was compared with combined treatment with salmeterol and fluticasone in patients with severe COPD; in this study, combined treatment was associated with a three-fold increased risk for CAP but differences in mortality were not observed. Moreover, Singh and co-workers [23] in a meta-analysis of randomised clinical trials with inhaled steroids as the intervention drug, have shown an increased risk of CAP with the use of inhaled steroids although it was not accompanied by a higher CAP-related mortality or overall mortality. In the same line, a recent meta-analysis [24] in which randomised, double-blind clinical trials carried out in patients with COPD were analysed, showed an increased risk for CAP associated with the use of inhaled steroids but when the effect on mortality was assessed, results were not statistically significant (OR = 0.86, 95% CI 0.68-1.09). Ernst et al. [10] reported a higher severity of CAP assessed by the need of in-patient care among patients previously treated with inhaled steroids, with a direct dose-hospitalization risk relationship. Moreover, the risk of hospitalization for CAP decreases progressively after withdrawal of inhaled steroid therapy. According to these data, inhaled steroids may be related to other physiopathological mechanisms that may act on the severity of CAP independently of the reduction of airway inflammation and the recruitment of neutrophils.

In the presence of this controversy, we here provide the results of a population-based study with more than half of the cases being treated in the outpatient setting. Although we found that on overall the treatment with inhaled steroids was associated to criteria of severity or worse prognosis, in terms of hospital admission, after adjusting by other severity-related factors, the use of inhaled steroids did not show an independent effect on the prognosis of CAP. Therefore, treatment with inhaled steroids seems to be a consequence rather than a cause of severity. However, it is very difficult to differentiate the severity of CAP from the severity of the underlying chronic pulmonary diseases because both diseases are closely linked. Nevertheless, in the group of patients with CB, but not in patients with COPD or patients with asthma, inhaled steroids seem to prevent hospitalization for CAP. In more severe diseases (patients with COPD) the protective effect of inhaled steroids disappears. The effect of inhaled steroids is thus modified by the type of underlying chronic respiratory disease.

The present results, however, should be interpreted taking into account some limitations of the study. In particular, and although the large study sample, we observed a small incidence of outcomes in terms of mortality and ICU admission. This is in agreement with the clinical course of the CAP [16], but it could affect the statistical power to detect some effect of inhaled steroid therapy in these outcomes. In addition, patients were followed for a short period of time and, therefore, only immediate prognosis or early outcome of the acute pneumonic episode was evaluated. Finally, the analyzed data correspond to patients assessed 12 years ago. During this period management of inhaled treatments and CAP may have changed in usual clinical practice.

In summary, this study found no evidence to support the hypothesis that previous use of inhaled steroids improves clinical outcomes in CAP patients with asthma or COPD. However, the use of inhaled steroids has a protective effect on hospital admission for CAP, but not in other clinical outcomes, in patients with CB. Our results are in contrast to some existing studies and, thus, further research is required in this controversial topic.
